# Population consequences of climate change through effects on functional traits of lentic brown trout in the sub-Arctic

**DOI:** 10.1038/s41598-021-94350-x

**Published:** 2021-07-27

**Authors:** Kim Magnus Bærum, Anders G. Finstad, Eva Marita Ulvan, Thrond O. Haugen

**Affiliations:** 1 Norwegian Institute for Nature Research, Fakkelgården, 2624 Lillehammer, Norway; 2 Department of Natural History, Centre for Biodiversity Dynamics, Norwegian University of Science and Technology, 7491 Trondheim, Norway; 3 Norwegian Institute for Nature Research, 7485 Trondheim, Norway; 4 Department of Ecology and Natural Resource Management, Norwegian University of Life Sciences, P. O. Box 5003, NO-1432 Aas, Norway

**Keywords:** Ecology, Climate sciences

## Abstract

Climate-induced plasticity in functional traits has received recent attention due to the immense importance phenotypic variation plays in population level responses. Here, we explore the effect of different climate-change scenarios on lentic populations of a freshwater ectotherm, the brown trout (*Salmo trutta* L.), through climate effects on functional traits. We first parameterize models of climate variables on growth, spawning probability and fecundity. The models are utilized to inform a dynamic age-structured projection matrix, enabling long-term population viability projections under climate and population density variation. Ambient temperature and winter conditions had a substantial effect on population growth rate. In general, warmer summer temperatures resulted in faster growth rates for young fish but ended in smaller size at age as fish got older. Increasing summer temperatures also induced maturation at younger age and smaller size. In addition, we found effects of first-year growth on later growth trajectories for a fish, indicating that environmental conditions experienced the first year will also influence size at age later in life. At the population level, increasing temperatures average (up to 4 °C increase in areas with mean summer temperature at approximately 12 °C) resulted in a positive effect on population growth rate (i.e. smaller but more fish) during climate simulations including increasing and more variable temperatures.

## Introduction

Climate change is expected to induce a variety of ecological responses through phenotypic plasticity^1^ and evolutionary adaptation^2^. These responses range from behavior alterations^3^ to changes in phenology^4,5^. Thus, it is not trivial to draw general conclusions about the direction or form of climate-change responses. However, an emerging trend with an increasing empirical evidence base for organisms with indeterminate growth seems to be related to the temperature-size rule^6^, where organisms exposed to warmer temperatures grow faster through ontogeny, but reach a smaller adult size^7–11^. These effects might be especially pronounced in systems where there are few opportunities for behavioral thermoregulation, and for ectotherms, as temperature and growth are directly connected. A less explored aspect of the temperature-size rule and global warming is if and how these changes in somatic growth will affect population dynamics. Somatic growth and size are key life-history traits across taxa as they often link directly with sexual maturity, survival, reproductive success and movement/migration^12–14^. Thus, understanding and forecasting how trait variation affects populations is crucial, because climate change will alter the phenotypic trait distributions. Recent studies on temperature dependent body size effects for aquatic ectotherms have shown a potentially large impact on the population dynamics^15–17^, and even on ecological interactions and food web responses^18^. However, as there are a multitude of population processes in the wild, there is still a large potential to explore and increase the understanding on how these act simultaneously to produce variations in population dynamics in a changing world. Interacting effects of population density and temperature might result in both synergistic^19^ and opposing^20^ effects on somatic growth in the wild, depending on habitat quality and food availability. It is also likely that a temperature effect on growth might come in addition to other effects of climate change, such as the effect of local precipitation patterns^21,22^. Therefore, to understand and forecast variation in population dynamics as a function of changing life history traits, modeling that captures the possible variation of these essential underlying mechanisms without being overly complex, is required.

Here, we explore how climate components affect population dynamics of lentic brown trout (*Salmo trutta*), via expected effects on functional traits. Through models based on variations within temperature and precipitation gradients, as well as density, we infer how individual fish size at age, fecundity and spawning norm might be affected. During climate simulations, all these life-history traits are allowed to interact and react to changes in the environmental variables, which dictate the parameter range in an age-structured matrix population model^23^ to forecast changes in population growth rate. Through the developed population tool, we explore the following research questions: How would an allopatric lentic brown trout population react to an increase in mean summer temperature, as well as increased variation in annual summer temperature, and how would added changes in winter conditions alter population dynamics further?

## Results

### Length at age

**Figure 1:**
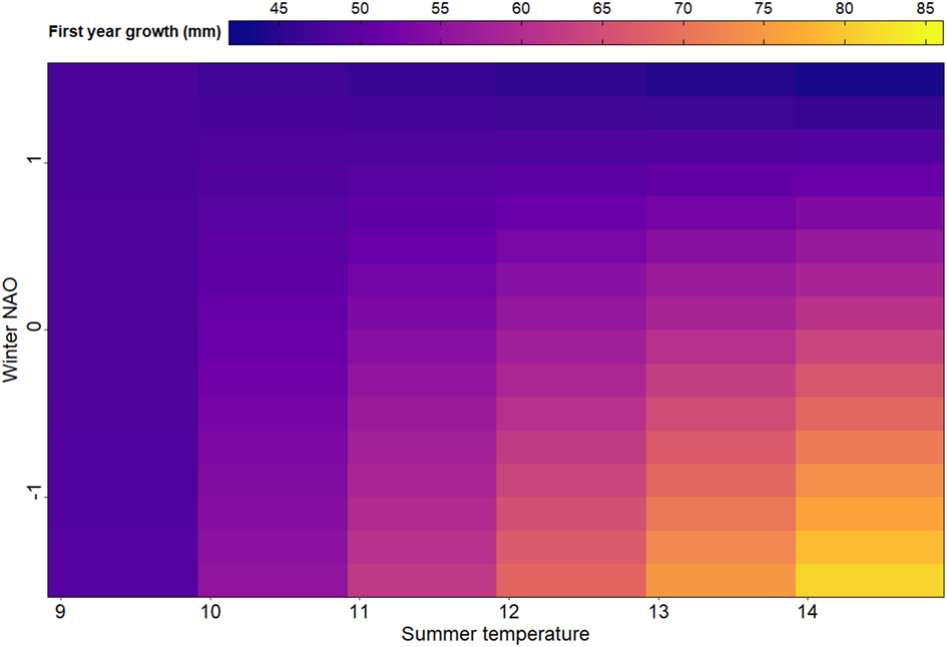
A contour plot of length at age 1 (~ first year growth), as predicted by the most supported model. Predicted length at age 1 in mm, as indicated by the variation in colour, is shown as functions of air summer temperature (x-axis) and winter NAO-index value (y axis). The figure has been made using the ggplot2-library^68^.

For length at age one, the model including an interaction effect between summer temperature and winter North Atlantic Oscillations-index^24^ (NAO, Table 1) got considerable support over the other candidate models. This model predicted that summer temperature had a positive effect on first year growth, but this was increasingly and most pronounced for declining and low winter NAO values (i.e., a tendency of colder and dryer winters) (Fig. 1). For high winter NAO values, the model predicted a general pattern of small length at age, and as the NAO index increased towards maximum, the growth was increasingly unaffected by variations in the summer air temperature (Fig. 1).

**Table 1:** Estimated model coefficients for the fixed effects in the most supported model for length at age 1 and length at age > 1, respectively. Numbers in parenthesis represent standard errors for the estimates.

Fixed effects	Length at age 1	Length at age > 1
	Model coefficients	Model coefficients
Intercept	13.35 (5.58)	102.90 (7.81)
Length at age 1	–	1.23 (0.04)
CPUE	–	− 0.64 (0.41)
NAO	22.33 (4.62)	6.60 (4.82)
Temp	2.81 (0.46)	5.47 (0.62)
Age	–	56.68 (2.01)
NAO*Temp	− 2.42 (0.39)	− 0.83 (0.38)
Age*NAO	–	0.52 (0.50)
Age*Temp	–	− 1.73 (0.18)
Observations	889	2503

**Figure 2:**
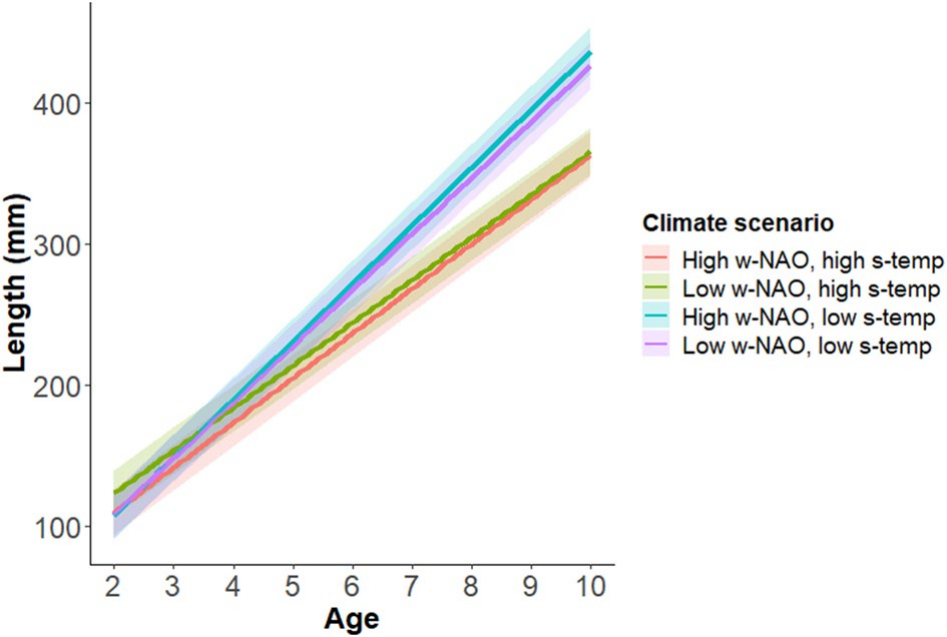
Predicted mean length in mm (y-axis) at age (x-axis) from the most supported growth model. The variation between the different lines illustrates differences in predicted mean length at age given four different combination of summer air temperature and winter NAO-index values. Low and high mean summer air temperature considered in the growth projections are 10.6 °C and 15.1 °C, respectively, while high and low w-NAO represents -0.82 and 1.85, respectively. Shaded areas show the standard deviation of the predicted mean length at age. The figure has been made using the ggplot2-library^68^.

For length at age greater than one, there were two candidate models that got considerable support over the other models in the selection procedure (model selection table can be seen in Supplementary information S1). Although the result of the two models differed only marginally, we chose to do model averaging over these two models to get a single set of representable parameter estimates to include in the population simulations. The parameter estimates can be seen in Table 1. In general, the model predicted a positive effect of first year growth and a negative effect of CPUE (capture per unit effort, used as a proxy for density), both effects lasting throughout the fish life span. Further, low average summer temperatures resulted in slightly smaller length at age < 3, but increased length for fish older than 3 (Fig. 2). The effect of winter NAO index on length at age was in general small, but being most pronounced during high summer temperatures (Fig. 2).

### Spawning probability

**Figure 3:**
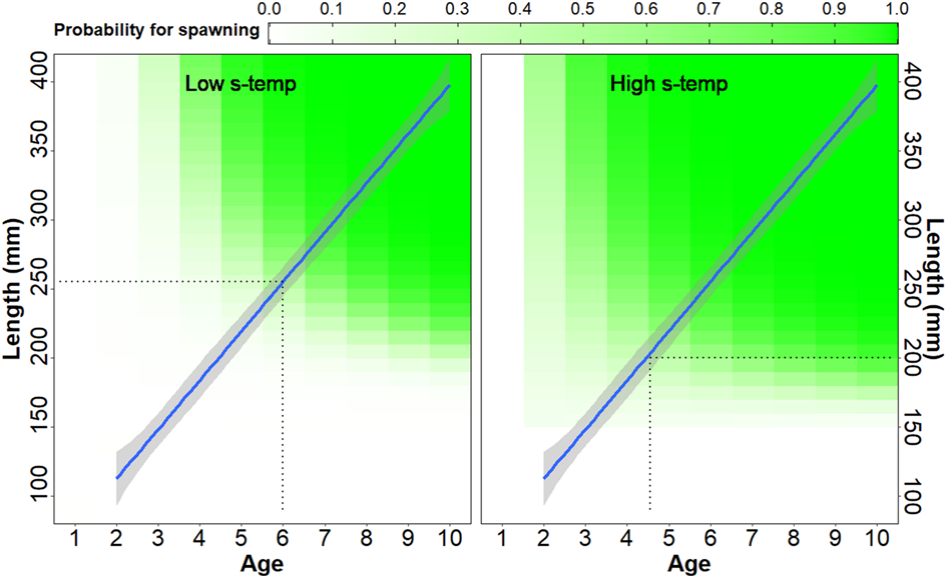
Contour plot representing variations in spawning probability (green shading) in relation to age (x-axis) and length in millimetres (y-axis). A value of 0.1 equals 10% probability of spawning, 0.2 equals 20% and so on. Left panel shows the spawning probability for lakes in the data experiencing low mean summer temperatures (10.8 °C), and right panel shows the spawning probability for lakes experiencing high mean summer temperatures (13.4 °C). Blue lines represent the estimated mean length at age across all the lakes and the corresponding shaded areas are 95% confidence bounds. The dotted lines indicate the approximate point of 50% probability to spawn in the two temperature scenarios. The figure has been made using the ggplot2-library^68^.

The most supported model for spawning probability included an effect of summer temperature in addition to the length-age interaction effect (Supplementary information S2). Low summer temperatures resulted the spawning probability reaction norm to shift up and right, so that fish tended to spawn at an older age and larger size compared to fish experiencing warmer summer temperatures (probability reaction norm shifting down and left, Fig. 3). Thus, if two hypothetical fish were to follow the same growth trajectory in two different lakes, one experiencing warm summer temperatures (~ 14 °C) and one experiencing low summer temperatures (~ 11 °C), the fish in the cold lake would reach a 50% probability of spawning at approximately 260 mm and 6 years of age while the fish in the warm lake would reach the same probability at approximately 200 mm and between age 4 and 5 (Fig. 3).

### Survival

Estimated annual survival rate (s) based on calculations from the descending catch curve (age 5–10) was estimated to be 0.496 (standard error = 0.04).

### Climate scenarios and population projections

**Figure 4:**
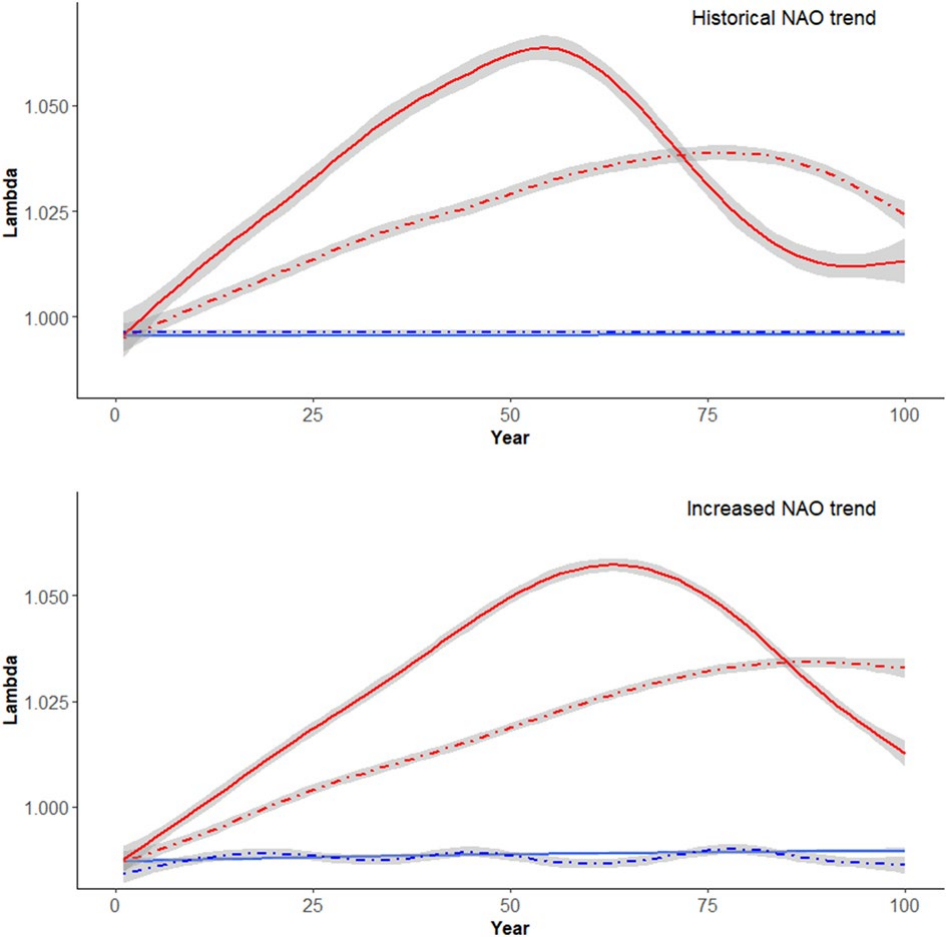
Estimated lambdas, interpreted as instantaneous population growth rate, (y-axis) as a function of time (x-axis) from the projection matrix model. Blue solid lines represent a status quo scenario with no temperature trend, blue dashed lines represent scenarios with no increase in the mean temperature, but with increased annual temperature variation, the red lines represent a climate scenario where annual mean summer temperature increases with 0.04 °C throughout the time frame and dot-dashed red lines represent expected lambdas during an annual summer temperature increase of 0.02 °C as well as increased annual temperature variation. All lines represent smoothed mean trends from 100 iterations of the respective climate scenario. Shaded areas represent the 95% confidence interval around the smoothed mean. Upper panel visualize projections for summer temperature scenarios with no particular trend in the NAO-index values (a proxy for winter conditions, following historical values for the last 20 years), and lower panel visualize the same summer temperature scenarios as above, but also including a trend of increased NAO-index values (a proxy for warmer and more snowy winter conditions). The figure has been made using the ggplot2-library^68^.

The climate scenarios with an increase in mean air summer temperature (mean annual increase of 0.04 °C) revealed a rather large effect on lambda (Fig. 4, upper panel). The rise in temperature induced an increasingly positive population trend until about 50 years out in the 100-year scenario, equivalent to an increase of mean summer temperature of 2 °C, where the model predicted a lambda ranging between 1.06 and 1.07. This would mean an annual increase in population size of up to 7%. However, from 50 to 100 years (2 °C to 4 °C increase in mean summer temperature), the potential for population growth was slightly reduced and seemingly stabilizing on a lambda value around 1.01. The climate scenario where annual mean summer air temperature increased with 0.02 °C, as well as allowing for larger annual variation in temperature, also induced a positive population trend. The potential of population growth had, however, a more gradual increase than was expected in the warmer climate scenario. Here, the highest rate of population increase was expected around year 75 in the simulation (~ 1.5 °C increase in temperature, lambda ranging between 1.03 and 1.04), and resulting in a lambda of approximately 1.03 after 100 years of climate change.

The climate scenarios where we also allowed for a positive trend in winter NAO-index values accompany the summer air temperature scenarios (i.e. also simulating warmer and wetter winter conditions), changed the above-mentioned expectation only slightly (Fig. 4, lower panel). For the scenario where summer temperatures had similar mean and variation as contemporary summer temperatures, there was an expected negative population trend (lambda between 0.99 and 0.98). The scenarios including a mean annual increase of 0.04 °C and a mean annual increase of 0.02 °C as well as larger variation between years did not change the respective maximum lambdas too much, but rather delayed the expected population projections compared to the scenarios with no expected change in winter trends. Larger annual variation in summer temperature by itself did not have a large effect on long-term population dynamics, however the combination of warmer winters with more precipitation (still usually as snowfall in the area) and increased annual variation in mean summer temperatures produced more oscillating population sizes (Fig. 4).

## Discussion

Our results show that changes in summer temperatures and winter conditions, and thus also size at age and spawning probability norm, can have profound population-level consequences for the studied sub artic brown trout communities. The relatively large changes in predicted instantaneous population growth rates were mostly apparent during climate scenarios with an increase in mean summer temperature, visible even at relatively conservative increases in temperature. In general, the climate-warming simulations forecasted increasing population sizes (Fig. 4), where individuals obtained smaller size at age, but also spawned at earlier age and smaller size compared to scenarios with no temperature increase. The apparent bell shaped curves of the expected instantaneous growth rate as a function of time for scenarios with increasing temperature (Fig. 4), are likely a result of density regulation in the simulations (stock-recruitment relationship, as well as density effect on growth), gradually reducing the high potential for population growth. The population growth potential is, however, always above 1 and therefore the populations are always increasing in size during the time and temperature frame of our simulations. The situation for sub-Artic brown trout in face of global warming thus seems to be more much more positive, compared to populations in warmer regions^25^. Our findings corresponds with a comparable study which also found a positive effect of temperature on population growth of pike (*Esox lucius* L.) in the Windermere lake system, United Kingdom, as a result of the fish following temperature-size rules during temperature warming^26^. Interestingly, Vindenes et al*.*^26^ also found that variable temperature regimes had little effect on the population dynamics compared to a stable increase in temperature, which indeed seems to be reflected in our results as well. We do, however, see a tendency of oscillating population sizes if more variable summer temperature regime is accompanied by warmer winters with more precipitation. The latter is likely still to fall as snow in most of the areas that our data is extracted from and would thus also likely produce a shorter growth season as snow accumulates during winter.

The effect of winter conditions in general (as interpreted from NAO-index values), had most effect on young individuals, where index-values associated with warmer winters and more snow resulted in smaller sizes at age. At the population level, this resulted in a tendency of declining population size (λ < 1) for the scenario with unchanged summer temperatures, while populations experiencing warmer summers still experienced increase in population size. The trend of reduced somatic growth as a result of warmer winters with more snow is expected as this would negatively affect the length of the effective growth season^27–29^. Nonetheless, the environmental effect on size at age 1 (i.e. experienced growth between age 0 and 1) was still influential in determining the intercept of the growth trajectory for later ages. In other words, if a fish experienced poor growth conditions early in life (low summer temperature and/or high winter NAO), this seemed to carry over to reduce size at age throughout ontogeny. This response is in line with the results from a recent study of temperature effects on Eurasian perch (*Perca fluviatilis*) where larger body size at age also was associated with high juvenile growth rates^30^. The results from Huss et al*.*^30^ contrasts the temperature size-rule^6^, as a smaller body size for adults would be expected as juvenile growth increased. In that perspective, our results diverge from Huss et al*.*^30^, as we indeed see evidence for the temperature size-rule with increased juvenile growth and a reduced adult size (Figs. 2, 3). The differences is, however, that a reduction or increase in effective growth season duration early in life might lower or heighten the expected growth trajectory through life (change the intercept of the growth trajectory), while the temperature still affects the slope of the growth trajectory. These are likely two different mechanistic processes in play, one acting according to the length of growth season (intercept), and perhaps also the mismatch between food availability and temperature, while the other might be related to metabolic effects of temperature (slope). This explains how experienced growth early in life can be influential in determining realized size at mature age, even though the growth trajectory stays rather unchanged through ontogeny. It also adds to the notion that body size-temperature relations are probably not general and simple, but rather complex with proximate explanations that vary according to the specific situations and organisms^31,32^.

The brown trout included in our study likely experienced water temperatures that mostly were below or just at the optimal temperature for individual growth, never exceeding thermal tolerances for either fry or adult fish^33^. Our model projections therefore also reflect general expectations for similar sub-Artic populations, whereas populations now experiencing temperatures closer to thermal limits for growth and survival, are likely to be more negatively affected^34^. It is, however, interesting to observe that expected individual growth of adults is reduced as temperatures in theory moves towards more optimal conditions for growth (mean air summer temperature of approx. 10 °C to approx. 15 °C, Fig. 2). This is likely, at least partly, due to interactions between food-resources/density and thermal regimes^19,20,35,36^, reducing the capacity of laboratory-based growth-models for accurately predicting observed growth patterns in the wild.

It should be noted that due to restricted data available for the survival estimates, we were not able to explore how age-specific survival rates might change as functions of different environmental properties. We rather choose to fix the survival rates in the population projection process, which perhaps gave more marginal projections compared to natural situations as survival might vary according to e.g., growth trajectory, winter conditions, temperature and density^37–42^. Furthermore, an accurate disentanglement, understanding and reassemble of all functional traits and environmental drivers in play to determine the population dynamics is a daunting task. For example, maturation norms and growth trajectories are likely very intertwined, where both traits jointly affect each other^43^. Our model approach is a simplification of such processes, based on best knowledge and observed mechanistic relationships between focal traits and environmental drivers. Furthermore, as our results are based on observed correlative relationships, we do not have a full causal explanation for the observed trends. For instance, we do not have detailed data on seasonal habitat utilization of the fish, nor variation in actual water temperature, but rather clear indications of trait variations as function of changes in air temperature. Consequently, we do not have information concerning the proximate cause of the temperature effect, which could both be related to metabolic constrains as well as for example food availability. Both examples are associated with differences in for example individual growth^20,44^. This sets some limitations to the range of temperatures where the model projections are likely to be representative (e.g. not for populations where temperatures are much higher than range observed in this study, as discussed above), but is also promising as air temperatures usually are more accessible across scales. Previous studies have found little support for large variation in thermal-related performance in brown trout^21,45^. As a consequence, the models can easily be reparametrized across larger geographical scales where fish data exist, but lake-specific environmental data lacks. This will further increase the understanding of population dynamics across an even wider range of temperatures than explored in this study.

In conclusion, our study shows that regionally down-scaled climate-warming scenarios are likely to have a positive impact on sub-arctic brown trout population sizes within the first half century, followed by a half century of declined or levelling-off population growth for high or intermediate summer temperature increase, respectively. The population structure changed towards smaller individuals that matured earlier at smaller sizes under both climate warming scenarios. Changes in winter climate towards increasingly warmer and wetter (yet, snow condition) slightly moderates and delay these population trajectories. Interestingly, the modelled sub-arctic brown trout populations performed less optimal when run under contemporary climate conditions. An interesting question that arises, and that calls for further research, is how robust these populations with many small sized individuals are to withstand other relevant anthropogenic disturbances such as the introduction of alien fish species or changes in the habitats?

## Material and methods

### Sampling and data

**Figure 5:**
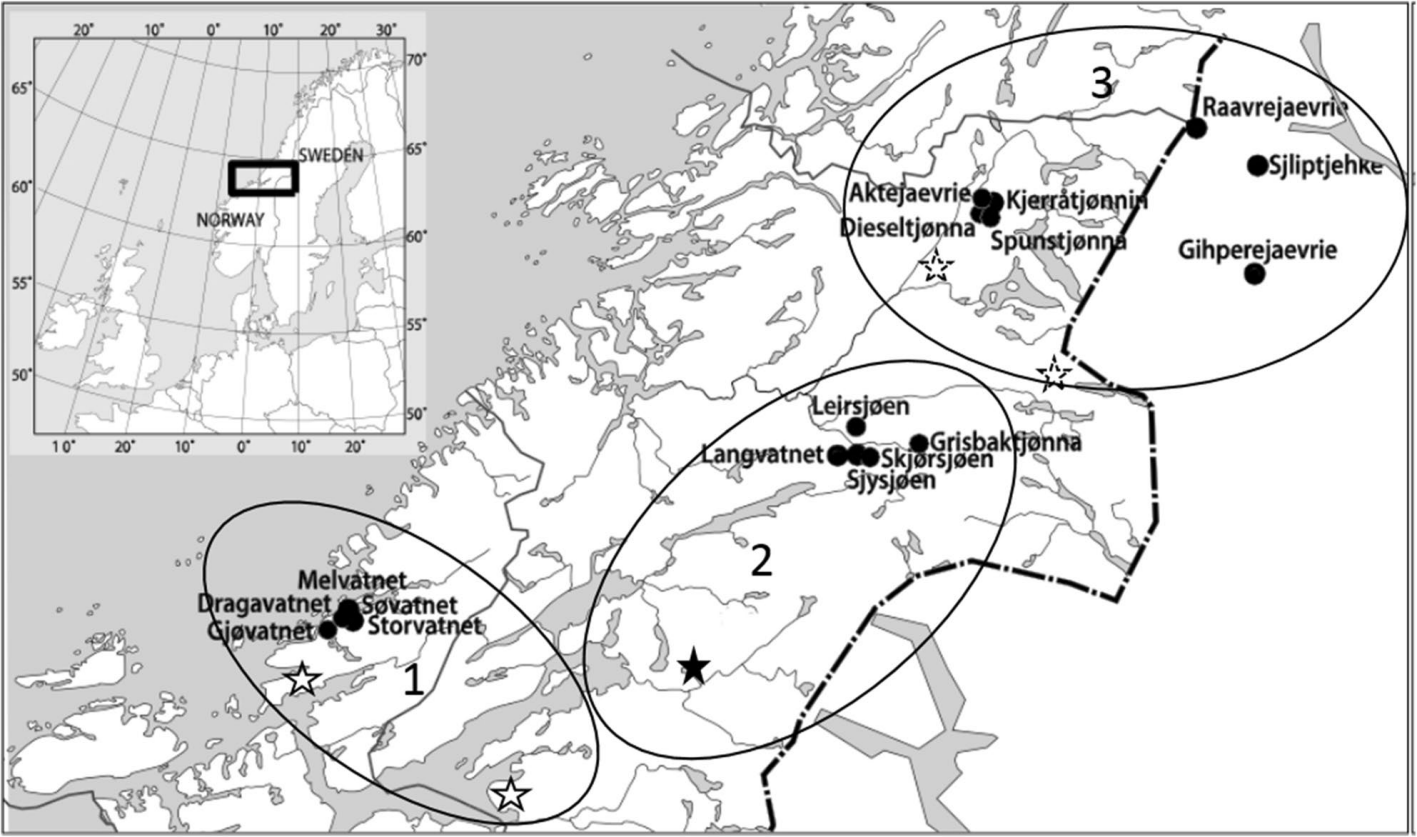
Study lake positions (filled dots) and names. Unfilled large circles connects the different lakes with the most representative weather stations (stars) in the area (in terms of altitude, vegetation zones and landscape). The dashed line constitute the national border between Norway and Sweden. The figure was produced using Adobe illustrator.

The data consist of gillnet catches of brown trout (N = 5733, caught during 2008–2009) from 21 lakes situated along an altitudinal gradient (30 m above sea level, m.a.s.l.-800 m.a.s.l.) in mid-Norway and Sweden (Fig. 5). The lakes were sampled within three main types of vegetation zonation in the catchment area that ranged from the southern boreal to the alpine zone. The lowland lakes were situated in the southern boreal zone dominated by coniferous woodland and forest, but there were also large areas of alder (*Alnus* sp.) as well as some broad-leaved deciduous woodland. Average annual and July air temperature are 4–6 and 12–16 °C, respectively^46^. Middle boreal catchment area is dominated by coniferous woodland, forest and mires. Average annual and July air temperature are, respectively, 2–4 and 8–12°C^46^. Vegetation around the high altitude lakes were dominated by bilberry (*Vaccinium myrtillus*), grass heaths and dwarf birch (*Betula nana*) scrub, with annual and July air temperature of − 2 to 0 and 6–12°C^46^. The clustering of lakes within vegetations zones can be seen in Fig. 5. The epilimnetic water temperature across a sample of the lakes in the altitudinal gradient in this study seems to be within the general trends in the air temperature^47^.

All lakes were sampled using standardised gillnet series consisting of single mesh nets (25 × 1.5 m) with mesh sizes 12.5, 16, 19.5, 24, 29 and 35 mm^47^. Three nets were linked together making chains with alternating mesh sizes in order to represent all mesh sizes at different depths in each lake at each sampling. This gillnet series catches brown trout with a slight bias in favour of larger individuals^48^ that was assumed similar in all lakes. The nets were distributed along the shoreline, and the lakes were fished during summer, with different effort (i.e., number of gillnet series) depending on lake size. Weight per unit catch effort (CPUE) based on total weight of the brown trout catch per 100 m^2^ gillnet area per night was used as a proxy for biomass density. Since, differences in environmental conditions across lakes cause large variations in body size and hence per capita resource demands, biomass was considered a better measure of population density than number of individuals for among-lake comparisons. Length (total length, mm) and weight (g) at catch were measured for every individual in the full data set. Age, sex, maturation status and back-calculation of length-at-age was undertaken for a randomly selected representative subset (N = 889) of the data. Growth and spawning probability ogives^49^ were modeled based on this subset. Scale samples and otoliths were taken and used for age determination, of which scales were used primarily, and scales were used for back-calculation of growth^50^. Distance between the annuli was measured, and a direct proportional relationship between the length of the fish and the scale radius was assumed^51^. If the scales were difficult to read, which was the case for more slow-growing individuals from the low altitude lakes were the annuli were less distinct, otoliths were used for determining the age. As we did not have complete records of water temperature, area and time specific summer air temperature and precipitation measurements were obtained from an online database (www.eklima.no, Norwegian Metrological Institute). The database contained historical weather data from the closest representative (i.e., corresponding in distance, altitude and operational period) weather stations to the respective lakes (Fig. 5). This resulted in overlapping temperature and precipitation regimes for some of the lakes as there were in total five different weather stations that were most representative within the area containing the 21 lakes. Further, as there was some variation in how complete the different measurements were within years, we also had to calculate the sum of summer precipitation for a shorter period of the summer compared to the average mean air temperature. Both measurements still being good proxies for experienced summer conditions in the bulk of the growth season. The effect of temperature and precipitation was thus derived from the spatio-temporal variation in observations between these five weather stations, where the historic temporal variation corresponds to recorded climate components relevant to years for the back calculated age of the individual fish in the specific lakes (resulting in a total of 29 distinct measurements, see variation in Table 2) Epilimnetic water of lakes usually reflects warming trends in air temperature well, however hypolimnetic temperature variation might not be very correlated to the air temperature. Yet, changes in air temperature might indeed influence the thermal stratification of a lake and thus the environment and conditions for a fish^52^. There are good reasons to believe that most of the lakes in our study obtained some sort of thermal stratification during the summer season. Nonetheless, we chose not to model air to water temperature for the few measurements of water temperatures we had, and extrapolate this relationship to the full spatio-temporal resolution of the data. The rationale for this was threefold: (1) We were interested in exploring potential effects and relationships of easily available climate components, such as air temperature, simplifying the model concept; (2) we did not have access to detailed data on lake bathymetry so that hypothetical modeled air-to-water relationships would be rather uncertain; (3) we had no detailed information on how the brown trout was distributed in the water column during the summer period in study lakes. However, compared to similar lakes, there are reasons to believe that brown trout mainly feed and stay in the upper six meters of the water column, as well as epibenthic areas with high invertebrate abundances^53,54^, where both areas often are overlapping and highly influenced by the air temperature.

**Table 2:** Description of candidate variables used in the model selection process determining the most supported model for individual growth of brown trout.

Variables	Description	Numerical summery (mean, std. deviation, min and max) rounded to nearest decimal
Temperature	Year and area specific mean air temperature (°C) during 15th of May until end of September (obtained from the closest representative weather station)	11.8, 1.3, 9.4, 15.1
Summer precipitation	Year and area specific sum of precipitation (mm) from May until end of July (obtained from the closest representative weather station at www.eklima.no)	203.7, 38.1, 102.3, 371.4
CPUE	Lake specific catch per unit effort obtained from gillnets, used as an indication for abundance	4.1, 3.2, 0.6, 16.9
NAO	Year specific North Atlantic Oscillation-index during winter season (December–March) for all areas. Interpreted as an indicator of winter conditions. Low (negative) values usually leads to cooler and drier conditions (cold but less snow) in Norway and high values brings warmer and wetter weather to Norway (often meaning more snow in mid to high altitude areas)	0.3, 0.9, − 0.8, 1.9
Age	Age of the specific fish in growth season	3.2, 1.8, 1, 10

### Data analysis and model descriptions

#### Overall process

**Figure 6:**
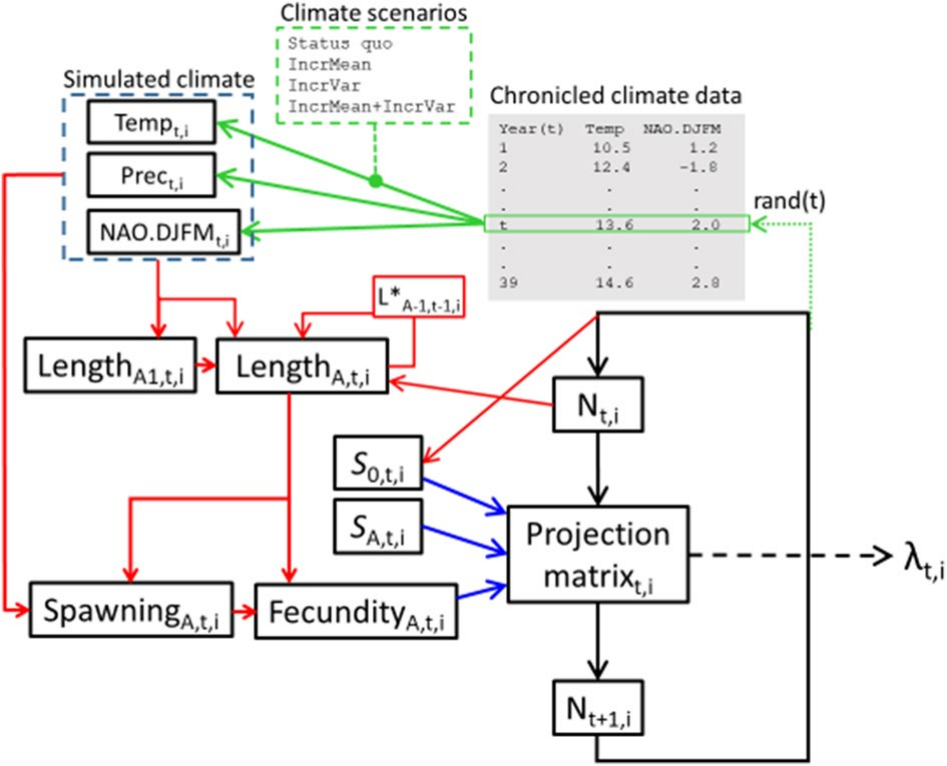
A schematic overview of the processes involved in our model-setup. Red lines indicate drivers and connections acting on individual life history traits, blue lines indicates traits driving the population model and green lines indicates links to climate variables. In short, existing area and time specific climate data on summer precipitation (Prec) and mean summer air temperature (Temp), as well as time specific data on winter NAO-index (recorded NAO values during December, January, February and March, NAO.DJFM), were used to parameterize models for length at age 1 and length at age > 1, as well as spawning probability at age. Length at age 1 was allowed to affect length at age > 1, and in the simulations achieved length at age > 1 was also influenced by the achieved length the previous year (L*). Length at age and spawning probability, both defined by climate variables, interacted in defining how many eggs a female was likely to produce (i.e. fecundity). Survival from eggs to small juvenile fish was based on a stock-recruitment relationship, where the stock was defined by the results from the population model (expected number of fish). Expected number of fish across all ages was also allowed to affect length at age > 1. The model parameters was used to simulate long term population dynamics, where we also varied expected temperature change scenarios (steadily increasing mean temperatures and temperature variation, respectively, as well as a combination of the two latter scenarios). The populations long term rate of increase (λ) was inferred using the age structured population matrix model.

We used linear mixed model approaches to parameterize environmental effects on key life history traits for brown trout. Specifically: Length at age was parameterized as function of the environment (e.g., summer temperature, population density, winter NAO and summer precipitation). Spawning probability were modeled as functions of individual length and age. We also allowed either the age effect or length effect on spawning probability to vary with temperature or summer precipitation. Individual fecundity (number of eggs produced) was predicted as a function of length and spawning probability. Annual survival estimates from age 1 and up was accessed using catch curve analysis, while first year survival was estimated based on a stock-recruitment function. The estimated parameters were utilized to feed an age structured matrix projection model^23^, enabling long-term population viability projections in an changing environment (see overview in Fig. 6). Although there are several choices of population models that might be utilized for inferring the population dynamics, such as IBMs^55^ and IPMs^56^, the age structured matrix model was deemed especially suited to model our systems as they are highly seasonal (with very reduced growth during winter) and thus producing a clear age structure in the data. Further description of the various modeling approaches are described below. All statistical analyses was done in R^57^.

#### Size at age

Data inspections prior to model development showed length at age to be surprisingly linear within the size and age distribution in our data (i.e. no obvious signs of asymptotic growth for fish in any of the sampled lakes). Length (L) was thus explored using a linear mixed effects model approach with the lme4-package^58^. Denoted, length for individual *j* in population *i* (L_ij_) could thus be expressed as:

$${L}_{ij}={\sum\limits_{k=1}^{p}}{\chi }_{ijk}{\beta }_{k}$$

Here, β = (β_1_, …, β_p_)^T^ is px1 vector (one column matrix) of unknown regression parameters, χ_i_^T^ = (χ_i1_, …, χ_ip_) ∈ ℝ^p^ is the explanatory variables of interest (*k* + *p* < *n*). ${L}_{ij}$ was assumed normally distributed. p is the number of explanatory variables included. Further, as the brown trout in this dataset stays (at least) one year in their natal tributary after hatching before they migrate to the lake, we choose to model individual size at age one and size at subsequent ages as two different processes. The variables considered in the candidate models were summer air temperature, summer precipitation, NAO-index (see extended variable description in Table 2). For size at age > 1, age was always included as a variable, and we also tested models including an effect of CPUE and first year growth on subsequent growth trajectories. Multiple candidate models where the different environmental effects were allowed to vary with age were constructed (Supplementary information S1). Population ID and individual ID were included as nested random effects in all candidate models exploring size at age > 1, and population ID was included as a random effect for the models exploring size at age 1. The most supported models were selected based on AIC-values^59^. During the population simulation the variation in the predictions attributed to the random effect(s) was treated as random noise, and not explicitly included in the simulations.

#### Spawning probability

Brown trout is an iteroparous species, however under normal food conditions and harsh winters in Norway it might not spawn every year following maturity. Accordingly, we modelled likelihood of spawning at age, derived from the number of female individuals that was going to spawn the following autumn, rather than probability of maturation at age. Aging effects on spawning probability was included in the modelling as skipped-spawning individuals (i.e., mature females that skip spawning episodes, sensu Rideout and Tomkiewicz ^60^) were coded as non-spawners in the analysis. Probability of spawning (*P*) was calculated based on a maturation-ogives approach^61^, utilizing generalized linear mixed effects models in the lme4-package^58^. Binomial models as two-dimensional ogives, *o*(*A, L*) were considered in the model selection. Here, *A* and *L* represent age and length, respectively. In addition, we also explored how these ogives might change due to either a temperature effect, summer precipitation effect, or a measure of fish abundance (CPUE) including either as an additive effect in some candidate models (see Supplementary information S2). Population ID was always included as a random effect. In general, the probability of spawning could thus be described as:

$${\mathrm{Pr}\left(spawning\right)}_{ij}={\beta }_{0i}+{\beta }_{1i}{A}_{ij}+{\beta }_{2i}{L}_{ij}+{\beta }_{3i}{A}_{ij}{L}_{ij}+{\beta }_{4i}{x}_{1i}+{a}_{i}+{\varepsilon }_{ij}$$

where βs represent coefficients under estimation, *A*_*ij*_ = age of individual *j* in population *i*, L = individual length, *x*_1_ represent a lake-specific environmental variable (if present in the candidate model, either summer temperature, CPUE or precipitation), *a*_*i*_ is the estimated random lake-specific intercept and *ε*_*ij*_ is the random residual variation assumed normally distributed on logit scale. The most supported model was selected based on AICc-values^59^.

#### Fecundity

Female fecundity (i.e., number of eggs per female) was predicted as a function of female length (mm) and two constants based upon published values for brown trout from Norway (*F* = *e* ^log(*l*)*2.21–6.15^)^62^ multiplied by the probability of spawning (*P*) at size and age.

#### Survival

Annual survival rates (s) for fish age ≥ 1 were based on estimations from catch-curve slopes utilizing the Chapman-Robson function in the FSA-package^63^. The survival was estimated based on descending catch curves, i.e., where numbers of caught individuals decreased as a function of age in the catch. Based on this slope we can derive an instantaneous mortality rate (*Z*), and from this the annual survival rate could be estimated from *S* = e^-*Z*^. Due to a restricted number of populations available for survival rates, the survival was estimated across all population. As it is unlikely that S would be constant across all age classes we choose to make age specific survival rates, S_a_, where the S_1_ (survival from age one to age two) was reduced, and S_3-5_ was slightly increased whereas all other S_a_ = S. The respective reduction and increase are described more in detail below. Survival rates for age 0–1, S_0_, was based on a stock-recruitment function (see further description under “*Climate scenarios, calibration and population projections*”).

#### The projection matrix

Population projections were derived utilizing an age-structured matrix population model^23^ in the popbio-package in R^64^. Changes in the age structure and abundance of brown trout was modelled from *N*_*t*+1_ = ***K***(*E,N,t*)*N*_*t*_ or rather:

$${\left[\begin{array}{c}{N}_{1}\\ {N}_{2}\\ \vdots \\ \vdots \\ {N}_{{a}_{max}}\end{array}\right]}_{t+1}=\left[\begin{array}{ccccc}{f}_{1}\left(L,P,{N}_{t}\right){s}_{0}\left({E}_{t}\right)& {f}_{2}\left(L,P,{N}_{t}\right){s}_{0}\left({E}_{t}\right)& \cdots & \cdots & {f}_{{a}_{max}}\left(L,P,{N}_{t}\right){s}_{0}\left({E}_{t}\right)\\ {s}_{a}& 0& \cdots & \cdots & 0\\ 0& {s}_{a}& \cdots & \cdots & 0\\ \vdots & \vdots & \vdots & \vdots & \vdots \\ 0& 0& 0& {s}_{a}& 0\end{array}\right]\times {\left[\begin{array}{c}{N}_{1}\\ {N}_{2}\\ \vdots \\ \vdots \\ {N}_{{a}_{max}}\end{array}\right]}_{t}$$

where *N*_t_ is the abundance of brown trout across all age classes *a* = 1,…, *a*_*max*_ at year *t*. Census time is chosen so that reproduction occurs at the beginning of each annual season. *f*_*a*_ is the fecundity at age *a* (i.e., the number of offspring produced per individual of age *a* during a year). More specifically, *f* varies according to *f*(*L,P*,*N*), where variations in *L* (length) and *P* (probability to spawn) in turn is defined by climate variables and the number of individuals *N*. *s* is a constant and represent the survival probability of individuals from age *a* to age *a* + 1, and *a*_*max*_ is the maximum age considered in the model. *a*_*max*_ was set to 10 years in the simulations, as was also was the age of the oldest fish in the aged subset of the data (see frequency table in Supplementary information S2). Although varying between systems, the maximum age observed and simulated also corresponds to expected maximum age found in other systems in Norway^65^. S_0_ is a function of *E,* the numbers of eggs laid, where the relationship is determined by a stock-recruitment function.

Consequently, ***K***(*E,N,t*), the Leslie matrix, is a function of N and *E*. In each time step, the survival of individuals in age class *a*_*max*_ is 0, whereas individuals at all other ages spawn and experience mortality as defined above. From the Leslie matrix ***K***, we can infer the population’s long-term rate of increase, λ, from the dominant eigenvector of the matrix^23^.

#### Climate scenarios, calibration and population projections

To explore the population effects of changes in summer air temperature or winter conditions we simulated different 100-years climate-change scenarios for a single lake, which included variations the climate variables in focus. The first scenario represented a status quo setting. Here, annual average summer air temperatures were randomly drawn from a normal distribution with mean and standard deviation from observed summer air temperatures from 1998–2009 in the study area. The second climate scenario randomly assigned temperatures as in scenario one, as well as allowing for more and more fluctuating annual summer temperatures as time progressed. This was done by adding a random variable t (~ *N*(0,0.03) times the number of the specific year (i.e., 1–100) in the 100-years climate change scenario. The third climate scenario, drew annual summer temperatures as in the first scenario, but included an increase in the average air summer temperature by 0.04 °C each year (i.e., 4 °C in total for the 100-year-scenario which is close to the expected mean increase in regional temperature following the regionally down-scaled RCP8.5 IPCC scenario^66^). The fourth climate scenario included an average summer temperature increase of 0.02 °C each year (close to the expected average temperature increase following the regionally down-scaled RCP4.5 IPCC scenario^66^), as well as allowing for more and more fluctuating annual summer temperatures as time progressed (as in scenario two). For all climate scenarios above, annual winter NAO-values was randomly drawn from a uniform distribution between − 1.5 and 1.5.

We also simulated a second set of climate change scenarios, where summer temperatures were as described in the four scenarios above, however in all these scenarios we also included a trend of higher winter NAO values (meaning a general trend of warmer winters with more precipitation/snow in the study area, as predicted by the down scaled climate scenarios^66^). This was done by letting annual NAO-values be drawn from a random normal distribution with mean = 0.5, and standard deviation of 0.5.

During the calibration process for the simulations, we altered the age specific survival estimates S_1_ and S_3-5_ so that average lambdas for the status quo climate scenario was relatively stable and close to 1 (i.e. no large changes in population size) based on 100 iterations of a 100 year-climate scenario. Specifically, S_1_ = S*0.6 and S_3-5_ = S*1.2, which is also assumed to be within the realistic range of survival rates for the specific age classes in the focal populations. S_0_ was derived from a stock recruitment function, and was thus allowed to vary as a function of density in the population. Specifically, from the total egg number (*E*_*t*_) at year *t* and the number of one-year olds at year *t* + 1 (*N*_1,t+1_) the stock-recruitment function could be estimated by fitting a Shepherd function^67^:

$${N}_{1,t+1}=\frac{a{E}_{t}}{{\left(1+b{E}_{t}\right)}^{c}}$$

where a = 0.04, b = 0.0000003 and c = 3.5. *E* is number of eggs deposited during *t*-1 spawning season, estimated as the total fecundity. The estimated *N*_1,t+1_ was used to estimate first-year survival (*s*_0_) from:

$${s}_{0,t}=\mathrm{ln}\left({E}_{t-1}\right)-\mathrm{ln}\left({N}_{1,t}\right)$$

All 100-years scenarios were simulated with 100 iterations to extract the variation in the expected population projections. CPUE in the simulations was included as a dynamic variable in the growth model, recalculated through the matrix projection model for each time step, i.e. year. Length at age, spawning probability and fecundity was predicted for each time step (i.e. pr year) as described above. The spawning probability did however not vary annually according to changes in the environment but was predicted according to the mean values of the environmental variables across all years the climate scenario. However, for climate scenarios with increasing mean temperature over time, the expected spawning probability was a function of the gradual mean temperature increase. Thus, by allowing the spawning probability reaction norm gradually to follow changes in the temperature, as predicted from the spawning model, we allowed the populations to gradually adapt the reaction norm to the respective changes.

## Supplementary Information

Supplementary Information.

## References

[1] Przybylo R, Sheldon BC, Merila J (2000). Climatic effects on breeding and morphology: Evidence for phenotypic plasticity. J. Anim. Ecol..

[2] Bradshaw WE, Holzapfel CM (2006). Evolutionary response to rapid climate change. Science.

[3] Charmantier A (2008). Adaptive phenotypic plasticity in response to climate change in a wild bird population. Science.

[4] Wedekind C, Küng C (2010). Shift of spawning season and effects of climate warming on developmental stages of a grayling (salmonidae). Conserv. Biol..

[5] Visser ME, Both C (2005). Shifts in phenology due to global climate change: The need for a yardstick. Proc. R. Soc. B Biol. Sci..

[6] Atkinson D (1994). Temperature and organism size: A biological law for ectotherms. Adv. Ecol. Res..

[7] Berrigan D, Charnov EL (1994). Reaction norms for age and size at maturity in response to temperature: A puzzle for life historians. Oikos.

[8] van Rijn I, Buba Y, DeLong J, Kiflawi M, Belmaker J (2017). Large but uneven reduction in fish size across species in relation to changing sea temperatures Glob.. Change Biol..

[9] Gardner JL, Peters A, Kearney MR, Joseph L, Heinsohn R (2011). Declining body size: A third universal response to warming?. Trends Ecol. Evol..

[10] Ohlberger J (2013). Climate warming and ectotherm body size: From individual physiology to community ecology. Funct. Ecol..

[11] van Gils JA (2016). Body shrinkage due to Arctic warming reduces red knot fitness in tropical wintering range. Science.

[12] Peters R (1983). The Ecological Implications of Body Size.

[13] Stearns SC (1992). The Evolution of Life Histories.

[14] Wootton RJ (1998). Ecology of Teleost Fishes.

[15] Ohlberger J, Edeline E, Vollestad LA, Stenseth NC, Claessen D (2011). Temperature-driven regime shifts in the dynamics of size-structured populations. Am. Nat..

[16] Vindenes Y, Langangen Ø, Winfield IJ, Vøllestad LA (2016). Fitness consequences of early life conditions and maternal size effects in a freshwater top predator. J. Anim. Ecol..

[17] Lindmark M, Huss M, Ohlberger J, Gårdmark A (2018). Temperature-dependent body size effects determine population responses to climate warming. Ecol. Lett..

[18] Lindmark M, Ohlberger J, Huss M, Gårdmark A (2019). Size-based ecological interactions drive food web responses to climate warming. Ecol. Lett..

[19] Crozier LG, Zabel RW, Hockersmith EE, Achord S (2010). Interacting effects of density and temperature on body size in multiple populations of Chinook salmon. J. Anim. Ecol..

[20] Bærum KM, Haugen TO, Kiffney P, Moland Olsen E, Vøllestad LA (2013). Interacting effects of temperature and density on individual growth performance in a wild population of brown trout. Freshw. Biol..

[21] Bærum KM, Vøllestad LA, Kiffney P, Rémy A, Haugen TO (2016). Population-level variation in juvenile brown trout growth from different climatic regions of Norway to an experimental thermal gradient. Environ. Biol. Fishes.

[22] Siepielski AM (2017). Precipitation drives global variation in natural selection. Science.

[23] Caswell H (2001). Matrix Population Models.

[24] Hurrell JW, Kushnir Y, Ottersen G, Visbeck M, Hurrell JW, Kushnir Y, Ottersen G, Visbeck M (2003). An overview of the North Atlantic oscillation. The North Atlantic Oscillation: Climatic Significance and Environmental Impact.

[25] Mitro MG, Lyons JD, Stewart JS, Cunningham PK, Griffin JDT (2019). Projected changes in Brook Trout and Brown Trout distribution in Wisconsin streams in the mid-twenty-first century in response to climate change. Hydrobiologia.

[26] Vindenes Y (2014). Effects of climate change on trait-based dynamics of a top predator in freshwater ecosystems. Am. Nat..

[27] Helland IP, Finstad AG, Forseth T, Hesthagen T, Ugedal O (2011). Ice-cover effects on competitive interactions between two fish species. J. Anim. Ecol..

[28] Shuter BJ, Finstad AG, Helland IP, Zweimüller I, Hölker F (2012). The role of winter phenology in shaping the ecology of freshwater fish and their sensitivities to climate change. Aquat. Sci..

[29] Borgstrøm R (2001). Relationship between spring snow depth and growth of brown trout, *Salmo trutta*, in an Alpine Lake: Predicting consequences of climate change. Arct. Antarct. Alp. Res..

[30] Huss M, Lindmark M, Jacobson P, van Dorst RM, Gårdmark A (2019). Experimental evidence of gradual size-dependent shifts in body size and growth of fish in response to warming. Glob. Change Biol..

[31] Angilletta MJ, Dunham AE (2003). The temperature-size rule in ectotherms: Simple evolutionary explanations may not be general. Am. Nat..

[32] Myrvold KM, Kennedy BP (2019). Seasonal variation in growth, consumption and growth efficiency in overwintering juvenile steelhead. Ecol. Freshw. Fish.

[33] Jonsson B, Jonsson N (2009). A review of the likely effects of climate change on anadromous Atlantic salmon *Salmo salar* and brown trout *Salmo trutta*, with particular reference to water temperature and flow. J. Fish Biol..

[34] Almodóvar A, Nicola GG, Ayllón D, Elvira B (2012). Global warming threatens the persistence of Mediterranean brown trout. Glob. Change Biol..

[35] Ayllón D (2019). Mechanistic simulations predict that thermal and hydrological effects of climate change on Mediterranean trout cannot be offset by adaptive behaviour, evolution, and increased food production. Sci. Total Environ..

[36] Sánchez-Hernández J, Cobo F (2019). Suboptimal growth among individuals of brown trout (*Salmo trutta*) in a temperate river. J. Fish Biol..

[37] Pepin P (1991). Effect of temperature and tize on development, mortality, and survival rates of the pelagic early life history stages of marine fish. Can. J. Fish. Aquat. Sci..

[38] Pauly D (1980). On the interrelationships between natural mortality, growth parameters, and mean environmental temperature in 175 fish stocks. J. Cons..

[39] Jensen AJ, Johnsen BO (1999). The functional relationship between peak spring floods and survival and growth of juvenile Atlantic Salmon (*Salmo salar*) and Brown Trout (*Salmo trutta*). Funct. Ecol..

[40] Jonsson N, Jonsson B, Hansen LP (1998). The relative role of density-dependent and density-independent survival in the life cycle of Atlantic salmon *Salmo salar*. J. Anim. Ecol..

[41] Cianfrani C, Satizábal HF, Randin C (2015). A spatial modelling framework for assessing climate change impacts on freshwater ecosystems: Response of brown trout (*Salmo trutta* L.) biomass to warming water temperature. Ecol. Model..

[42] Nater CR (2020). Size- and stage-dependence in cause-specific mortality of migratory brown trout. J. Anim. Ecol..

[43] Tréhin C (2021). Growth during the first summer at sea modulates sex-specific maturation schedule in Atlantic salmon. Can. J. Fish. Aquat. Sci..

[44] Elliott JM, Hurley MA (2000). Optimum energy intake and gross efficiency of energy conversion for brown trout, *Salmo trutta*, feeding on invertebrates or fish. Freshw. Biol..

[45] Forseth T (2009). Thermal growth performance of juvenile brown trout, *Salmo trutta*: No support for thermal adaptation hypotheses. J. Fish Biol..

[46] Moen A (1998). Nasjonalatlas for Norge: Vegetasjon. Statens kartverk.

[47] Ulvan E, Finstad A, Ugedal O, Berg O (2012). Direct and indirect climatic drivers of biotic interactions: Ice-cover and carbon runoff shaping Arctic char *Salvelinus alpinus* and brown trout *Salmo trutta* competitive asymmetries. Oecologia.

[48] Jensen JW, Hesthagen T (1996). Direct estimates of the selectivity of a multimesh and a series of single gillnets for brown trout. J. Fish Biol..

[49] Heino M, Dieckmann U, Godo OR (2002). Measuring probabilistic reaction norms for age and size at maturation. Evolution.

[50] Jonsson B (1977). A method for estimating fish length from otolith size. Rep. Inst. Freshw. Res. Drottningholm.

[51] Francis RICC (1990). Back-calculation of fish length: A critical review. J. Fish Biol..

[52] Adrian R (2009). Lakes as sentinels of climate change. Limnol. Oceanogr..

[53] Hegge O, Dervo BK, Skurdal J, Hessen DO (1989). Habitat utilization by sympatric arctic charr *Salvelinus alpinus* L. and brown trout *Salmo trutta* L. in Lake Atnsjø, south-east Norway. Freshw. Biol..

[54] Hesthagen T, Jonsson B, Ugedal O, Forseth T (1997). Habitat use and life history of brown trout (*Salmo trutta*) and Arctic charr (*Salvelinus alpinus*) in some low acidity lakes in central Norway. Hydrobiologia.

[55] DeAngelis DL, Grimm V (2014). Individual-based models in ecology after four decades. F1000prime Rep..

[56] Merow C (2014). Advancing population ecology with integral projection models: A practical guide. Methods Ecol. Evol..

[57] R: A language and environment for statistical computing. R Foundation for Statistical Computing, Vienna, Austria. URL https://www.R-project.org/ v. 3.6.3 (2020).

[58] Bates D, Maechler M, Bolker B, Walker S (2015). Fitting linear mixed-effects models using lme4. J. Stat. Softw..

[59] Burnham KP, Anderson DR (2002). Model Selection and Multimodel Inference: A Practical Information-Theoretic Approach.

[60] Rideout RM, Tomkiewicz J (2011). Skipped spawning in fishes: More common than you might think. Mar. Coast. Fish..

[61] Olsen EM (2004). Maturation trends indicative of rapid evolution preceded the collapse of northern cod. Nature.

[62] Gregersen F, Vøllestad LA, Olsen EM, Haugen TO (2009). Sibling-size variation in brown trout *Salmo trutta* in relation to egg size and stream size. J. Fish Biol..

[63] Ogle, D. H. FSA: Fisheries Stock Analysis. *R package version 0.4.44*.

[64] Stubben CJ, Milligan BG (2007). Estimating and analyzing demographic models using the popbio package in R. J. Stat. Softw..

[65] Jonsson B (1985). Life history patterns of freshwater resident and sea-run migrant brown trout in Norway. Trans. Am. Fish. Soc..

[66] Hanssen-Bauer, I. *et al.* Klima i Norge 2100: Kunnskapsgrunnlag for klimatilpasning oppdatert i 2015. *NCCS report: 2/2015. 204 pp. In Norwegian (region down-scaling based on climate models presented in the IPCC (2013) report)* (2015).

[67] Shepherd JG (1982). A versatile new stock-recruitment relationship for fisheries, and the construction of sustainable yield curves. ICES J. Mar. Sci..

[68] Wickham H (2009). ggplot2: Elegant Graphics for Data Analysis.

