# Supplementary material for: Population consequences of climate change through effects on functional traits of lentic brown trout in the sub-Arctic
Source: Sci Rep. 2021 Jul 27;11:15246. doi: 10.1038/s41598-021-94350-x (PMC8316365; doi:10.1038/s41598-021-94350-x)
Supplement: Supplementary file 1 — Supplementary Information. [file 41598_2021_94350_MOESM1_ESM.docx]

**Population consequences of climate change through effects on functional traits of lentic brown trout in the sub-Arctic*.***

**Kim Magnus Bærum*^,1^, Anders G. Finstad^2^, Eva Marita Ulvan^3^, Thrond O. Haugen^4^**

**1** Norwegian Institute for Nature Research, Fakkelgården, 2624 Lillehammer, Norway

**2** Norwegian University of Science and Technology, Department of Natural History, 7491 Trondheim

**3** Norwegian Institute for Nature Research, 7485 Trondheim, Norway

**4** Norwegian University of Life Sciences, Department of Ecology and Natural Resource Management, P. O. Box 5003, NO-1432 Aas, Norway

**Supplementary information**

**S1**: Ordered model selection table for the fixed effect structure for the candidate models for fish-length at age 1 and age > 1. Independent variables used are: Length after the first year of growth (LFG), age (A), lake specific catch weight per unit effort (WPUE) , summer precipitation (SP), summer temperature (ST) and winter NAO-index (WNAO). Population ID was included as random effect for models exploring size at age 1, while Population ID and individual ID were included as nested random effects in all candidate models for size at age > 1.

| **Candidate models** | **Df** | **ΔAIC** | |
| --- | --- | --- | --- |
| **Size at age 1** |  |  |  |
| ST + WNAO + ST:WNAO | 6 | 0.0 |  |
| ST + WNAO + SP + ST:WNAO | 7 | 6.5 |  |
| WNAO + ST | 5 | 34.8 |  |
| SP + ST + WNAO | 6 | 43.9 |  |
| WNAO | 4 | 47.7 |  |
| SP + WNAO | 5 | 51.3 |  |
| ST + SP + WNAO + ST:SP | 7 | 53.1 |  |
| ST | 4 | 95.6 |  |
| 1 (intercept only) | 3 | 95.7 |  |
| ST + SP + ST:SP | 6 | 96.0 |  |
| SP + ST | 5 | 101.9 |  |
| SP | 4 | 105.3 |  |
| **Size at age > 1** |  |  |  |
| LFG + A + WPUE + WNAO + ST + WNAO:A + WNAO:ST + A:ST | 12 | 0.0 |  |
| LFG + A + WPUE + WNAO + ST + WNAO:ST + A:ST | 11 | 1.4 |  |
| LFG + A + WPUE + WNAO + ST + LFG:A + WNAO:ST + A:ST | 12 | 6.8 |  |
| LFG + A + WPUE + ST + A:ST | 9 | 9.9 |  |
| LFG + A + WPUE + WNAO + ST + WNAO:ST | 10 | 113.0 |  |
| LFG + A + WPUE + ST | 8 | 120.4 |  |
| LFG + A + WPUE | 7 | 120.8 |  |
| LFG + A + WPUE + WNAO + ST | 9 | 121.0 |  |
| LFG + A + WPUE + WNAO | 8 | 121.7 |  |
| LFG + A + WPUE + SP + WNAO + ST + WNAO:ST | 11 | 122.4 |  |
| LFG + A + WPUE + SP + ST | 9 | 128.6 |  |
| LFG + A + WPUE + SP + WNAO + ST | 10 | 128.7 |  |
| LFG + A + WPUE + A:LFG | 8 | 128.9 |  |
| LFG + A + WPUE + SP | 8 | 130.6 |  |
| LFG + A + WPUE + SP + WNAO | 9 | 131.3 |  |
| LFG + A + WPUE + ST + SP + ST:SP | 10 | 137.9 |  |
| LFG + A + WPUE + ST + SP + WNAO + ST:SP | 11 | 138.3 |  |
| A + WPUE + WNAO + ST + A:ST + WNAO:ST | 10 | 546.4 |  |
| A + WPUE | 6 | 666.1 |  |
| A | 5 | 673.2 |  |

**S2**: Model selection table for candidate models for the spawning probability, based on AICc-values. Independent variables used are: Age (A), fish length (L), lake specific catch weight per unit effort (WPUE) , summer precipitation (SP) and summer temperature (ST). Population ID was included in all candidate models as a random intercept.

| **Candidate model** | **DF** | **ΔAIC** |
| --- | --- | --- |
| A + log(L) + log(ST) + A:log(L) | 6 | 0 |
| A + log(L) + log(SP) + A:log(L) | 6 | 2.8 |
| A + log(L) + WPUE + A:log(L) | 6 | 4.9 |
| A + log(L) + A:log(L) | 5 | 6.5 |
| A + log(L) | 4 | 18.3 |

**S2**: Table showing the total sample size by age, of the random sample of trout in the catch that was chosen for age determination.

| **Age** | **Sample size** |
| --- | --- |
| 1 | 10 |
| 2 | 65 |
| 3 | 135 |
| 4 | 169 |
| 5 | 220 |
| 6 | 147 |
| 7 | 90 |
| 8 | 31 |
| 9 | 17 |
| 10 | 5 |
